# The Yin and Yang of Tyrosine Kinase Inhibition During Experimental Polymicrobial Sepsis

**DOI:** 10.3389/fimmu.2018.00901

**Published:** 2018-04-30

**Authors:** Cassiano Felippe Gonçalves-de-Albuquerque, Ina Rohwedder, Adriana Ribeiro Silva, Alessandra Silveira Ferreira, Angela R. M. Kurz, Céline Cougoule, Sarah Klapproth, Tanja Eggersmann, Johnatas D. Silva, Gisele Pena de Oliveira, Vera Luiza Capelozzi, Gabriel Gutfilen Schlesinger, Edlaine Rijo Costa, Rita de Cassia Elias Estrela Marins, Attila Mócsai, Isabelle Maridonneau-Parini, Barbara Walzog, Patricia Rieken Macedo Rocco, Markus Sperandio, Hugo Caire de Castro-Faria-Neto

**Affiliations:** ^1^Laboratório de Imunofarmacologia, Instituto Oswaldo Cruz, FIOCRUZ, Rio de Janeiro, Brazil; ^2^Walter Brendel Centre, Department of Cardiovascular Physiology and Pathophysiology, Klinikum der Universität, Ludwig Maximilians University München, Munich, Germany; ^3^Laboratório de Imunofarmacologia, Instituto Biomédico, Universidade Federal do Estado do Rio de Janeiro, Rio de Janeiro, Brazil; ^4^Institut de Pharmacologie et de Biologie Structurale, IPBS, Université de Toulouse, CNRS, UPS, Toulouse, France; ^5^Laboratory of Pulmonary Investigation, Carlos Chagas Filho Institute of Biophysics, Federal University of Rio de Janeiro, Rio de Janeiro, Brazil; ^6^Laboratório de Genômica Pulmonar, Faculdade de Medicina, Universidade de São Paulo, São Paulo, Brazil; ^7^Laboratorio de Farmacologia, Carlos Chagas Filho Institute of Biophysics, Federal University of Rio de Janeiro, Rio de Janeiro, Brazil; ^8^Laboratório de Pesquisa Clínica em DST e AIDS, Instituto Oswaldo Cruz, FIOCRUZ, Rio de Janeiro, Brazil; ^9^MTA-SE “Lendület” Inflammation Physiology Research Group, Department of Physiology, Semmelweis University, Budapest, Hungary

**Keywords:** sepsis, inflammation, dasatinib, Src tyrosine kinase, leukocyte trafficking

## Abstract

Neutrophils are the first cells of our immune system to arrive at the site of inflammation. They release cytokines, e.g., chemokines, to attract further immune cells, but also actively start to phagocytose and kill pathogens. In the case of sepsis, this tightly regulated host defense mechanism can become uncontrolled and hyperactive resulting in severe organ damage. Currently, no effective therapy is available to fight sepsis; therefore, novel treatment targets that could prevent excessive inflammatory responses are warranted. Src Family tyrosine Kinases (SFK), a group of tyrosine kinases, have been shown to play a major role in regulating immune cell recruitment and host defense. Leukocytes with SFK depletion display severe spreading and migration defects along with reduced cytokine production. Thus, we investigated the effects of dasatinib, a tyrosine kinase inhibitor, with a strong inhibitory capacity on SFKs during sterile inflammation and polymicrobial sepsis in mice. We found that dasatinib-treated mice displayed diminished leukocyte adhesion and extravasation in tumor necrosis factor-α-stimulated cremaster muscle venules *in vivo*. In polymicrobial sepsis, sepsis severity, organ damage, and clinical outcome improved in a dose-dependent fashion pointing toward an optimal therapeutic window for dasatinib dosage during polymicrobial sepsis. Dasatinib treatment may, therefore, provide a balanced immune response by preventing an overshooting inflammatory reaction on the one side and bacterial overgrowth on the other side.

## Introduction

Sepsis is a life-threatening systemic inflammatory condition which results in shock, multiple organ dysfunction, and eventually death (1, 2). It is characterized by a cytokine storm released from myeloid cells during an inadequate antimicrobial response to invading pathogens (3). Worldwide, more patients die due to sepsis-related complications than of breast and colorectal cancer together (4). The global incidence has been estimated to be 31 million including 6 million fatalities (5).

Neutrophil activation and invasion into inflamed tissue is a critical step in the host’s fight against an infection. Under normal circumstances, extravasation of neutrophils is tightly regulated by different receptors and ligands on both the endothelium and neutrophils (6). In order to transmigrate, neutrophils need to roll, adhere, and crawl along the activated endothelium to find a spot for extravasation. Following extravasation, neutrophils release cytokines like interleukin (IL)-1β, IL-6, and tumor necrosis factor (TNF)-α to attract more immune cells (7–9). In addition, they start to phagocytose pathogens. In sepsis, pathophysiologic processes are rather caused through an exuberant host response by immune cells against the invading microorganisms than through the direct effects of microbes itself (10). In this respect, a balanced immune response depends on regulatory mechanisms modulating the intensity of the immune response. The Src-family of tyrosine kinases (SFKs) are a group of signaling enzymes with diverse biological effects including, but not limited to, cell proliferation, survival, migration, and metastasis (11–13). SFKs are the largest family of cytoplasmic tyrosine kinases expressed in innate immune cells. The presence of those may vary between innate immune cells, with Hck, Fgr, and Lyn being the most prominently expressed SFKs in monocytes, macrophages, granulocytes, and dendritic cells (14). SFKs bind directly to the cytoplasmatic tail of activated integrins and are responsible for the majority of protein phosphorylations involved in integrin outside-in signaling. Various studies using knockout mice or inhibitors demonstrated the importance of SFKs in host defense and inflammation (15–19), including adhesion and transmigration during leukocyte recruitment (20). Because of these findings, tyrosine kinase inhibitors, originally designed for cancer therapy, have been studied for their role as immune-modulating drugs. Dasatinib, a multi-kinase inhibitor with strong effects on SFKs, acts on both Abl- and Src-family tyrosine kinases (21), and is currently used in patients with chronic myeloid leukemia and acute lymphoblastic leukemia with Philadelphia positive chromosome (Ph+) (22, 23). Besides its effect on malignant cells, dasatinib decreases systemic TNF-α production after LPS injection in a Src and Bruton’s tyrosine kinase dependent fashion (24) and reduced lung injury in a dose-dependent manner (25). Additionally, dasatinib treatment reduced chemokine secretion by neutrophils and bone marrow-derived macrophages, suggesting that SFKs are also critical regulators of chemokine secretion in myeloid cells (26). As immune responses to pathogens prevent their dissemination and favor their elimination by the host, there is a concomitant risk of exaggerated immune responses, which may lead to tissue and organ damage. Thus, we hypothesized that immunomodulatory drugs balancing immune responses may be beneficial during systemic severe infection. To test this, we investigated the safety and efficacy of the tyrosine kinase inhibitor dasatinib during inflammation and sepsis. We show that dasatinib diminished the recruitment of leukocytes to the site of inflammation in the inflamed cremaster muscle model. In addition, in a model of polymicrobial sepsis, dasatinib treatment improved survival and sepsis severity in mice and reduced organ damage in a dose-dependent manner with an optimal dose for survival.

## Materials and Methods

### Animals

We used male Swiss Webster (SW) mice (25–30 g) from the Oswaldo Cruz Foundation breeding unit, Rio de Janeiro, Brazil. Animals were lodged at 22°C with a 12-h light/dark cycle and free access to food and water. For *in vivo* cremaster muscle experiments *Lyz2*GFP and *Hck^−/−^Fgr^−/−^Lyn^−/−^* (SFK-ko) mice on a C57Bl/6 background were used (27–29). These mice were maintained at the Walter Brendel Center for Experimental Medicine, Ludwig Maximilians Universität, Munich, Germany and accommodated in a barrier facility under SPF conditions. Mice used in the experiment were at least 8 weeks of age and of healthy appearance.

### Pharmacokinetic Analysis

**Figure 1:**
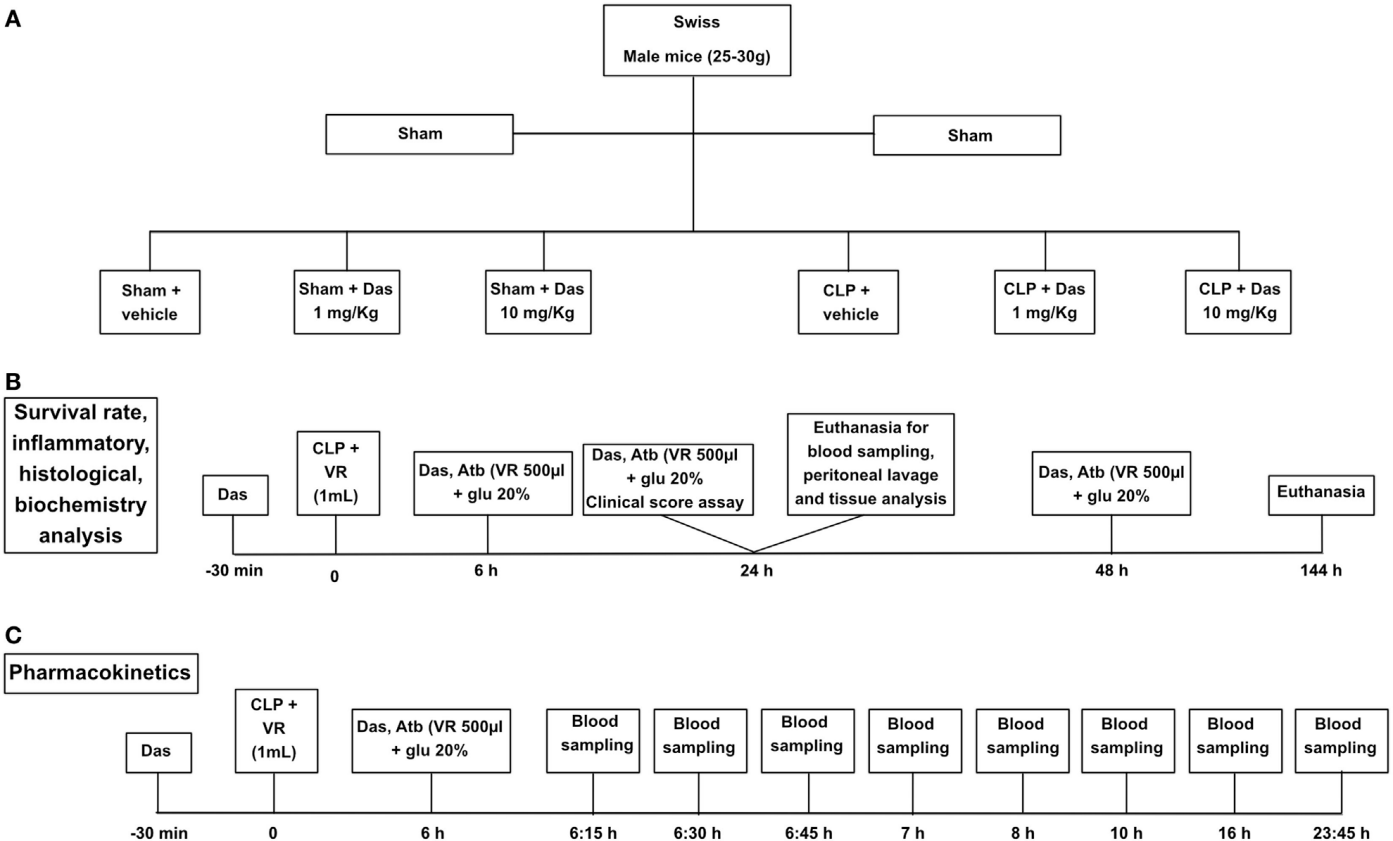
Experimental design. Group designation **(A)**, survival curve and inflammatory parameters **(B)**, and pharmacokinetics **(C)**. Sham, control group; CLP, cecal ligation and puncture; Das, dasatinib; Atb, antibiotic; VR, volemic reposition (500 µL); glu 20%, glucose 20%.

Pharmacokinetic evaluations were performed after the second administration of dasatinib (1 mg/kg). The administrations were made at the following time points: 30 min before CLP and 6 h after CLP. Blood samples were drawn at 0.25, 0.5, 0.75, 1, 2, 4, 8, 16, and 23.75 h (Figure 1C).

Dasatinib levels in plasma were determined using a validated high-performance liquid chromatography–tandem mass spectrometry method (HPLC–MS/MS). HPLC system (1200 series, Agilent Technologies, Germany) is connected with API 3200 triple quadrupole mass spectrometer (SCIEX, Toronto, ON, Canada) using multiple reaction monitoring (MRM). The MRM transitions monitored were *m/z* 488.2 → 401.3 for dasatinib, *m/z* 629.4 → 155.2 for internal standard.

### 3D Chemotaxis Assay

The analysis of migration in collagen gels was performed in μ-slide chemotaxis chambers (IBIDI, Planegg, Germany). A gel–cell mixture consisting of 3 × 10^5^ neutrophils in 1.5 mg/mL type I rat tail collagen (IBIDI) was applied to the middle channel of the 3D chamber and left at 37°C for 5 min for gelation. After application of 100 nM fMLP for 20 min at 37°C, time-lapse videos were recorded for 10 min using an Axiovert 200 M microscope (Zeiss, Jena, Germany) equipped with a Plan-Apochromat 10×/0.75NA objective, AxioCam HR digital camera, and a temperature-controlled environmental chamber. Migration tracks were analyzed offline with the Image J software. Single cell migration tracks and rose plots were generated using the IBIDI Chemotaxis software.

### Intravital Microscopy

We applied intravital microscopy in exteriorized inflamed cremaster muscle venules of *Lyz2* GFP and SFK-ko mice, as described (30). Briefly, mice were treated with intrascrotal injection of 500 ng TNF-α, 2 h prior to microscopy. Mice were then anesthetized with intraperitoneal (i.p.) injection of ketamine (125 mg/kg body weight, Ketalar; Parke-Davis, Morris Plains, NJ, USA), and xylazine (12.5 mg/kg body weight; Phoenix Scientific, Inc., St. Joseph, MO, USA). Thereafter, mice were placed on a heating pad to maintain body temperature, intubated, and the left carotid artery cannulated for blood sampling and systemic antibody administration. To maintain a neutral fluid balance, mice were given heparinized saline 0.2 mL/h i.v. throughout the experiment. Intravital microscopy was conducted on an upright conventional fluorescence microscope (Olympus BX51WI, Tokio, Japan) with a saline immersion objective (SW40/0.75 numerical aperture, Zeiss, Jena, Germany).

### Cremaster Muscle Preparation

The surgical preparation of the cremaster muscle was conducted as described (31). Shortly, after surgically opening the scrotum, the cremaster muscle was exteriorized and spread over a cover glass. The epididymis and testis were gently pinned aside giving full microscopic access to the cremaster muscle microcirculation. Experiments were recorded *via* a CCD camera system (CF8/1, Kappa, Gleichen, Germany) on a Panasonic S-VHS recorder and on hard-drive using virtual dub software. The cremaster muscle was superfused with thermocontrolled (35°C) bicarbonate-buffered saline. Postcapillary venules under observation ranged from 25 to 45 µm in diameter. Blood samples were taken during and after the experiment and WBC/neutrophil counts determined using ProCyte Dx Hematology Analyzer (IDEXX, Westbrook, ME, USA). Venular diameter, venular vessel segment length, and leukocyte rolling velocity were assessed using Fiji software (32). Venular centerline red blood cell velocity in the cremaster muscle preparation was measured during the experiment using a dual photodiode and a digital online cross-correlation program (Circusoft Instrumentation, Hockessin, DE, USA).

In a second set of experiments, the number of transmigrated cells was determined. For this approach, mice were treated as described above. After exteriorization, mouse cremaster muscles were dissected and fixed by 4% PFA (AppliChem GmbH, Darmstadt, Germany). Thereafter, cremaster muscle whole mounts were stained using Giemsa (Merck Millipore, Darmstadt, Germany) and the number of transmigrated cells/mm^2^ assessed using a Zeiss Axioskop 40 microscope with an oil immersion objective 100×, 1.25NA (Zeiss, Jena, Germany). Micrographic images are shown using an oil immersion objective 40×, 1.3NA (Zeiss).

### Dasatinib Treatment

*Lyz2GFP mice* received dasatinib (1, 10, or 20 mg/kg) by gavage in a volume of 100 μL/10 g methylcellulose. Control mice received methylcellulose alone. *Swiss* male mice received dasatinib (1 or 10 mg/kg) by gavage in a volume of 100 µL per animal 30 min before, 6 and 24 h after the induction of sepsis. Control animals received DMSO/saline solution (vehicle) same volume of dasatinb treatment. We based our treatment on dasatinib pharmacokinetics data reported by Ref. (33, 34). In acute experiments with end point at 24 h after cecal ligation and puncture (CLP) the animals received only the two first doses of the drugs (Figures 1A–C).

### CLP Model

Swiss mice were anesthetized by intraperitoneal injections of ketamine (100 mg/kg, Cristália) and xylazine (10 mg/kg, Syntec) 10–15 min prior to surgery. The cecal ligation was done below the ileocecal valve and the cecum was perforated four times with an 18G needle. A small amount of fecal material was squeezed from the holes before reinsertion of the cecum in the abdominal cavity. Volemic reposition was made with 1 mL of sterile saline subcutaneously. The animals received meropenem (10 mg/kg) diluted in salina with glucose at 20% intraperitoneally at 6, 24, and 48 h after CLP in 500 µL volume. Sham-operated animals constituted the control and received the same volume reposition and antibiotic treatment administered to CLP animals. After 24 h the animals were submitted to euthanasia using isoflurane (Cristália), and the peritoneal cavity was washed with PBS for colony-forming unit (CFU) analysis and total and differential leukocyte counting (Figures 1A,B).

### Biochemical Analysis

Mice were kept in a 12-h fasting with water *ad libitum*, and then blood was collected by cardiac puncture. Serum was separated by centrifugation and used for the quantification of albumin, creatinine, alanine, aspartate aminotransaminase. The quantifications were made using the dry chemistry methodology (Ortho Clinical—Johnson & Johnson) for biochemical parameters.

### Assessment of Sepsis Severity

At 24 h after CLP, mice were scored for severity of sepsis. In this assessment, higher scores reflect increased severity. Mice were scored based on the following variables: piloerection, curved trunk, alterations in gait, seizures, lethargy, respiratory rate, lacrimation, grip strength, feces alterations, body tone, and body temperature alterations [adapted from Ref. (35, 36)]. Each animal received a total score between 1 and 11 and was ranked as: 1–3 (mild sepsis); 4–7 (moderate sepsis); and 8–11 (severe sepsis). In our experimental conditions, most animals were ranked as moderate sepsis.

### Peritoneal Lavage

Briefly, mice were submitted to euthanasia 24 h after surgery using isoflurane (Cristália). The peritoneal cavity was washed with 3 mL of cold sterile saline in the laminar flow cabinet. The peritoneal washes were plated in Difco tryptic soy agar (TSA) (BD) for further analysis of bacterial growth through the count of CFU.

The peritoneal washes were also used for total cell count. Red blood cells were lysed using Turk solution (2% acetic acid) and total cell count was carried out using Neubauer chamber (Neubauer Improved). Differential leukocyte count was performed in cytocentrifuged smears stained with panotic (Laborclin). The supernatant was collected by centrifugation and stored at −20°C for further cytokine quantification.

### Cytokine and LTB_4_ Measurement

Tumor necrosis factor-α, IL-10, and IL-1β from the supernatant of peritoneal fluid or plasma were measured by enzyme-linked immunoabsorbant assay (ELISA, Duo set kit—R&D systems, Minneapolis, MN, USA) according to the manufacturer’s instruction. LTB_4_ was measured by enzyme immunoassay (EIA, Ann Arbor, MI, USA) according to the manufacturer’s instruction.

### CFU Counts

The number of CFU was determined in peritoneal lavage fluid, blood, and other organs that were diluted 1:10,000 and 1:1,000 and incubated under aerobic and sterile conditions on Difco TSA for 24 h at 37°C. The number of bacterial colonies were counted and expressed as CFU/mL.

### Histology

Histological analysis of omentum was performed as previously described (37, 38). Briefly, omentums were collected, fixed in 5% buffered formaldehyde and paraffin-embedded. Tissue sections (4 µm thick) were stained with hematoxyline and eosin for histomorphological analysis.

### Kidney, Small Intestine, and Liver Tissue Damage

The left kidney and the distal part of the right lobe of the liver were also removed after euthanasia. The tissues were fixed in 5% buffered formaldehyde, paraffin-embedded, and sections (4-µm thick) obtained. Liver sections were stained with hematoxylin–eosin, whereas kidney tissue was stained with periodic acid–Schiff reagent to visualize the basement membrane. 10 to 15 fields per section from random tubular regions of the renal cortex and liver parenchyma were captured at a magnification of 400×. Renal tubular damage was defined as tubular epithelial swelling, loss of brush border, vacuolar degeneration, and desquamation. A five-point, semi-quantitative, severity-based scoring system was used to assess each lesion parameter, graded as: 0 = normal tissue; 1 = 1–25%; 2 = 26–50%; 3 = 51–75%; and 4 = 76–100% of examined tissue.

In liver tissue, 10 fields per liver zone (central, lobular, and portal) were captured at a magnification of 400×. The ratio between sinusoidal cells and total cells was computed and expressed as percentage.

Image-Pro Plus 6.3 for Windows (Media Cybernetics, Silver Spring, MD, USA) was used for all analyses.

### DNA Measurement

Extracellular DNA was measured as an indicative of neutrophil extracellular trap (NET) formation. The DNA was quantified in the free cell peritoneal lavage fluid by using the Picogreen dsDNA kit (Invitrogen) according to the manufacturer’s instructions.

### DNAse Treatment

In some experiments, we also treated CLP or CLP+ dasatinib animals with DNase (5 mg/kg dissolved in saline solution enriched with 2 mM CaCl_2_, i.p., 1 h after CLP). The CFU was analyzed in peritoneal lavage at an earlier time point (3 h after CLP).

### Plasma Non-Esterified Fatty Acid (NEFA) Quantification

Plasma concentrations of the predominant NEFA—palmitic, oleic, linoleic, palmitoleic, and stearic acids—were assessed by a colorimetric assay (Zen-Bio, Inc.) (39).

### Phagocytosis Assay

Whole blood was collected from mice receiving dasatinib or vehicle. The assay was performed using the pHrodo *E. coli* bioparticles phagocytosis kit for flow cytometry (Invitrogen) according to the manufacturer’s instructions.

### Statistical Analysis

Data are represented as mean ± SEM and statistically analyzed by analysis of variance (one-way ANOVA) followed by Tukey and Student’s *t*-test. Survival curves and comparisons between curves were assessed using the Mantel–Cox log-rank test. *P values < 0.05 and ***P values < 0.001 were considered significant.

## Results

### Dasatinib Treatment Increased Rolling Velocity and Severely Impaired Neutrophil Adhesion *In Vivo*

**Figure 2:**
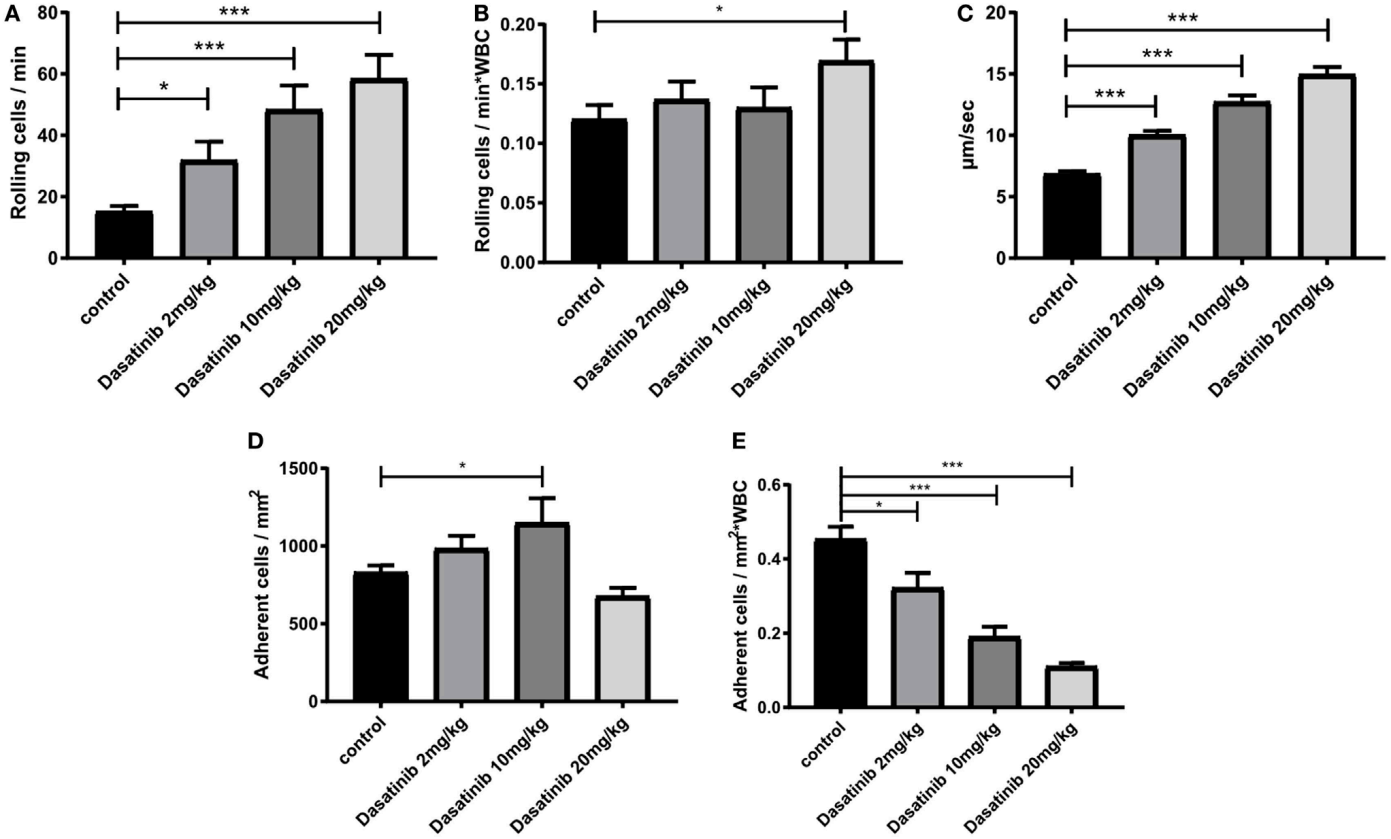
Dasatinib treatment increased rolling velocity and severely impaired neutrophil adhesion *in vivo. In vivo* leukocyte rolling was analyzed in rmTNF-α-stimulated venules of mouse cremaster muscles of Lyz^2^GFP mice pretreated orally with 2, 10, and 20 mg/kg dasatinib in methylcellulose, or with methylcellulose alone (control). Data are presented as mean ± SEM. **(A)** Number of rolling cells/min are shown. **(B)** Rolling flux fraction was calculated by the number of rolling cells/min normalized to the total WBC count. **(C)** Rolling velocities of neutrophils are displayed in micrometer per second. **(D)** Adherent cells were assessed over 1 min of observation. **(E)** Adhesion efficiency was calculated by the number of adherent cells/mm^2^ normalized to the total WBC count (*P < 0.05 and ***P < 0.001).

Dasatinib, a potent Src family kinase inhibitor is known to modu-late immune responses (40). Therefore, we set out to evaluate the effect of dasatinib on leukocyte recruitment in an *in vivo* model of TNF-α (2 h) induced inflammation of the mouse cremaster muscle using *Lyz2*GFP mice. Dasatinib (2, 10, or 20 mg/kg) was given orally 3 h prior to the exteriorization of the cremaster muscle. Observation of leukocyte rolling in cremaster muscle venules revealed a significant increase in the number of rolling leukocytes in the presence of dasatinib (Figure 2A). Because dasatinib is a broad-spectrum tyrosine kinase inhibitor, we additionally performed analysis of leukocyte recruitment in hck^−/−^ fgr^−/−^ lyn^−/−^ (SFK-ko) animals. In this model, all neutrophil-specific SFKs are deleted and, therefore, display a positive control for dasatinib specificity. We obtained comparable numbers of rolling cells/min in SFK-ko animals after TNF-α stimulation to 10 and 20 mg/kg dasatinib administration (Figure S1A in Supplementary Material). Because absolute numbers of rolling cells are influenced by changes in WBC count, we determined systemic leukocyte counts for each experiment and detected a dose-dependent increase in WBC counts following dasatinib application (Figure S1B in Supplementary Material). SFK-ko animals also showed an increase in the WBC count, following TNF-α stimulation, indicating that this is an SFK-dependent mechanism. We then assessed leukocyte rolling flux fraction, which is defined by the number of rolling leukocytes/min divided by the WBC count. Interestingly, this normalization reduced the observed increase in rolling (Figure 2B), indicating that dasatinib did not alter the relative number of rolling cells, but increased total circulating leukocytes. Next, we analyzed leukocyte rolling velocities and found a significant and dose-dependent increase in rolling velocity in the presence of dasatinib and in the SFK-ko mice (2 mg/kg 10.1 µm/s and 20 mg/kg 15.0 µm/s vs. 6.9 µm/s in WT control mice; Figure 2C; Figure S1C in Supplementary Material). This suggests that dasatinib inhibits Src kinase dependent intermediate activation of beta2 integrins, a process known to modulate rolling velocities in inflamed tissues (41). Interestingly, absolute number of adherent leukocytes in TNF-α (2 h) stimulated cremaster muscle venules of dasatinib-pretreated mice showed only minor changes (Figure 2D). However, after normalizing the number of adherent cells to changes in WBC revealed a severe and dose-dependent leukocyte adhesion defect (Figure 2E). This decrease was also visible in SFK-ko animals (Figure S1D in Supplementary Material). To exclude that these effects were due to changes in surface expression of rolling- and adhesion-relevant molecules, we performed FACS analysis of leukocyte surface molecules (Figure S1E in Supplementary Material). No major differences in surface expression could be detected for CD18, CD11a, CD11b, CD62L, PSGL1, CXCR2, and CD44.

Overall, these findings demonstrate that SFK inhibition by dasatinib significantly increases leukocyte rolling velocity and reduces leukocyte adhesion in inflamed postcapillary venules *in vivo*.

### Dasatinib Treatment Strongly Reduced Leukocyte Extravasation

**Figure 3:**
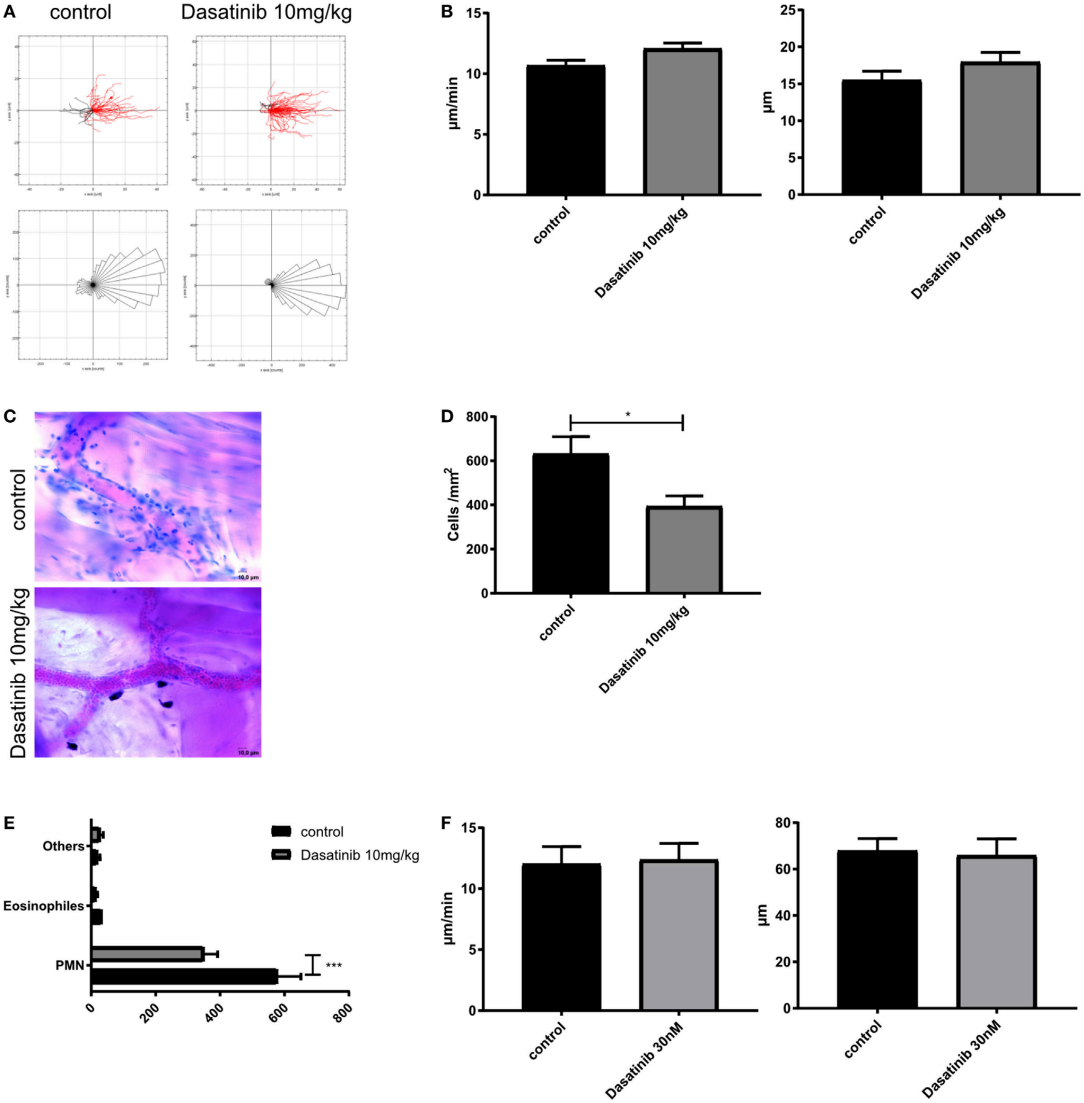
Dasatinib treatment strongly reduces leukocyte extravasation. *In vivo* crawling experiments were analyzed in rmTNF-α-stimulated Cremaster muscle tissue of Lyz^2^GFP mice, pretreated orally with 10 mg/kg dasatinib in Methylcellulose, or with Methylcellulose alone (control). Time-lapse movies over 15 min were performed and leukocytes visualized by their GFP signal. Extravasated leukocytes were analyzed with Giemsa staining in rmTNF-α-stimulated fixed cremaster muscle tissue of Lyz^2^GFP mice, treated orally with 10 mg/kg dasatinib in methylcellulose, or with methylcellulose alone. Data are presented as mean ± SEM. **(A)** Representative single cell migration tracks and rose plots for intraluminal crawling are displayed. Red lines indicate migration in, black lines migration against flow direction. At least 80 cells were analyzed for each strain **(B)** Evaluation of crawling velocity and Euclidian distance of crawling cells **(C)** Representative images of cremaster muscle whole mounts after Giemsa staining. Scale bar: 10 µm **(D)** Total number of extravasated cells/mm^2^ in muscle tissue in close proximity to a vessel. Star indicates significance over control. **(E)** Differential total cell counts of polymorphonuclear cells, eosinophils, and other cells. **(F)** Evaluation of migration velocity and Euclidian distance of crawling PMNs with or without dasatinib (*P < 0.05). Statistical analysis: Student’s *t*-test.

To extravasate into inflamed tissue, leukocytes need to crawl along the endothelial wall to find an appropriate spot for extravasation. We performed time-lapse fluorescence video microscopy in TNF-α (2 h) stimulated cremaster muscle venules and tracked GFP-fluorescent crawling leukocytes in control or dasatinib (10 mg/kg)-treated Lyz2GFP mice. In contrast to previous observations of neutrophil 2D migration in Zigmond chambers (42), dasatinib had almost no detectable effect on 2D neutrophil crawling *in vivo* (Figure 3A). No significant differences in crawling velocity or Euclidean distance were observed (Figure 3B), indicating that those cells which adhere in the presence of dasatinib display no further migration defect.

The crossing of the vascular wall is the last step in the leukocyte adhesion cascade. To quantify extravasation, we performed Giemsa staining of fixed cremaster muscle tissues of control and dasatinib (10 mg/kg) treated mice after TNF-α stimulation and counted perivascular leukocytes (Figure 3C). Quantification of extravasated cells revealed a significant inhibition of leukocyte extravasation by dasatinib compared to control animals (394 vs. 632 cells/mm^2^, Figure 3D). Likewise in SFK-ko mice, the number of extravasated cells was decreased to a similar degree (430 cells/mm^2^). This further strengthens our hypothesis of dasatinib acting mostly on SFKs during leukocyte recruitment. A more detailed analysis of leukocyte subtypes crossing the vessel wall showed that dasatinib mainly inhibited neutrophil extravasation (Figure 3E). In contrast to intraluminal crawling, leukocyte migration in the interstitium occurs in a 3D environment and is integrin-independent and eventually also SFK independent. We, therefore, performed *in vitro* 3D migration experiments to investigate the effects of dasatinib on integrin-independent migration. Indeed, analyzing migration of isolated leukocytes in a 3D collagen gel matrix revealed no alteration of leukocyte migration behavior in the presence of dasatinib (Figure 3F; Figure S1F in Supplementary Material). The Euclidean distance as well as their migration velocity was unchanged. This indicates that SFKs, similar to leukocyte integrins (43), are dispensable for interstitial migration once leukocytes managed to overcome the vascular barrier.

### Dasatinib Dose-Dependent Effect on Survival and Severity of Sepsis After CLP

**Figure 4:**
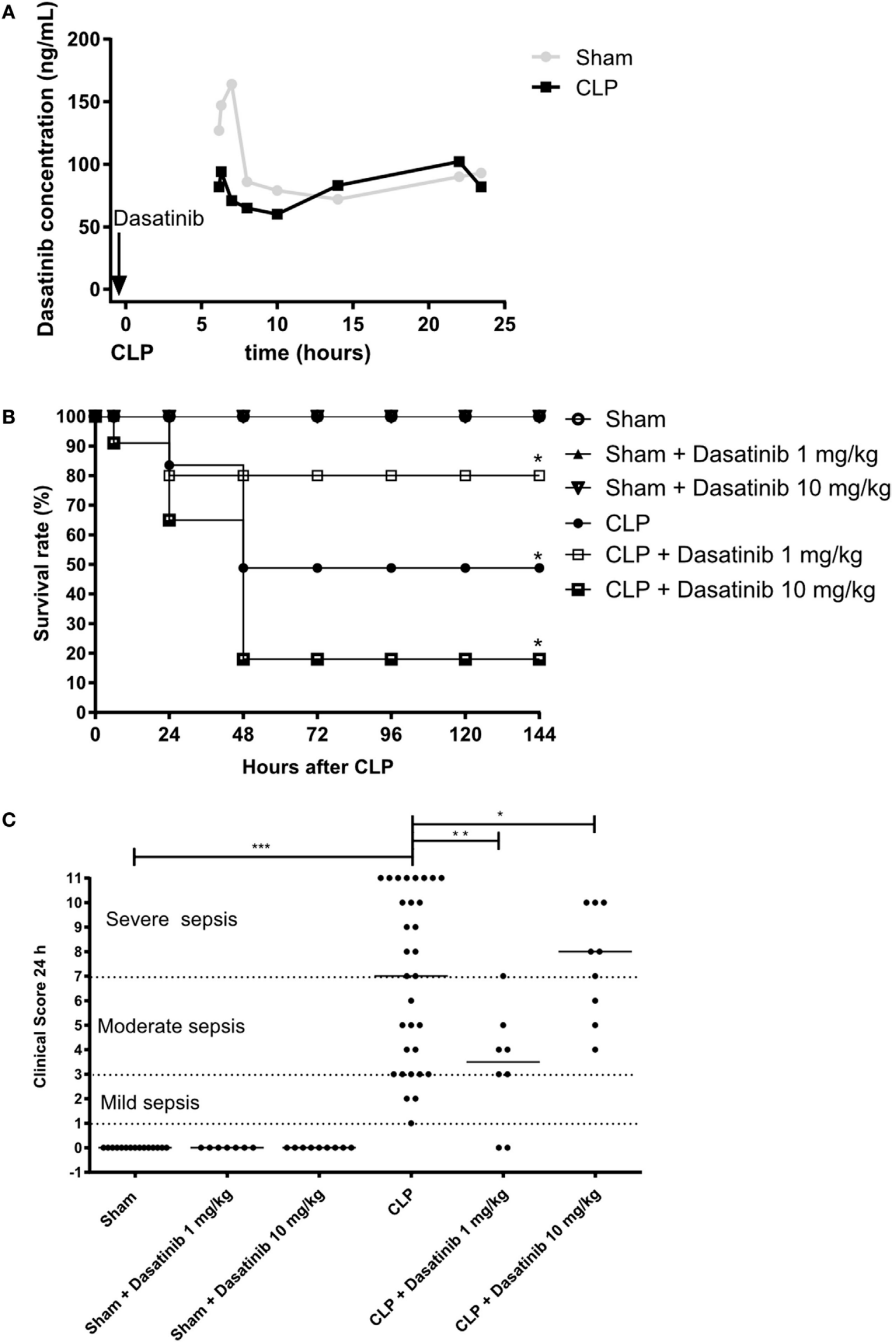
Dose-dependent effect of dasatinib on survival and severity of sepsis after cecal ligation and puncture (CLP). *Swiss* mice were submitted to CLP. Sham-treated animals were used as control. Data are presented as mean ± SEM. **(A)** Dasatinib was given orally 30 min before and 6 and 24 h after CLP. Dasatinib concentration of blood samples taken at indicated timepoints is displayed. **(B)** The survival rate was quantified for 7 days (144 h) in sham mice, untreated animals or each treated with dasatinib at 1 or 10 mg/kg dosage. **(C)** Clinical score was assessed 24 h after CLP. Each dot represents one animal. 1–3 points in the clinical score corresponds to a mild sepsis, 4–7 points corresponds to a moderate sepsis, and 8–11 points corresponds to severe sepsis. The animals were treated with dasatinib 1 or 10 mg/kg orally 30 min before, 6 and 24 h after CLP procedure. At least two independent experiments were performed. Statistical analysis: one-way ANOVA followed by Tukey *P < 0.05, **P < 0.01, and ***P < 0.001, for Figure 3B and Mantel–Cox log-rank test *P values < 0.05, for Figure 3C. The number of animals per group range from 3 to 4 for pharmacokinetics, 7 to 31 for clinical score, and 10 to 11 per group from mortality.

Our *in vitro* and *in vivo* findings described above suggest a potential role of dasatinib treatment on the outcome of sepsis. We, therefore, tested dasatinib administration (1 and 10 mg/kg) in the CLP model of induced sepsis. Our first step was to evaluate the bioavailability of dasatinib by measuring its concentration in the plasma of septic animals. For that, we performed pharmacokinetics analyses and compared the plasma concentration of 1 mg/kg dasatinib after administration to sham or CLP animals (Figure 4A). Septic animals had lower peak values of the drug than treated sham animals but their plasma levels remained at pharmacological levels up to 24 h after administration. Of note, plasma concentrations in both animals remained markedly above the concentration (14.9 ng/mL) able to inhibit 90% of phosphorylation of pBCR-ABL protein (34). We next induced polymicrobial sepsis using the CLP model to test for survival and clinical scores in septic mice. Sham-treated animal showed 100% survival rate after 7 days. Following CLP, we observed a survival rate of 50% after 7 days in control animals, with the highest mortality observed between day 1 and 2 after CLP. In contrast, administration of dasatinib at 1 mg/kg protected the animals from lethal sepsis following CLP (Figure 4B). Dasatinib at 1 mg/kg administered 30 min before and 6 and 24 h after CLP resulted in an 80% survival rate 7 days after CLP. Interestingly, a higher dose (10 mg/kg) of dasatinib had an opposite effect, with a mortality rate increasing to 85%.

The beneficial effect of low dasatinib doses was also detected in the severity score. As shown in Figure 4C, 1 mg/kg dasatinib improved severity scores compared to untreated CLP animals and resulted in only moderate sepsis scores. Again 10 mg/kg dasatinib reversed this effect. Sham-treated animals did not present any sign of disease.

### Low Dosage of Dasatinib Decreased Organ Dysfunction in Septic Animals

**Figure 5:**
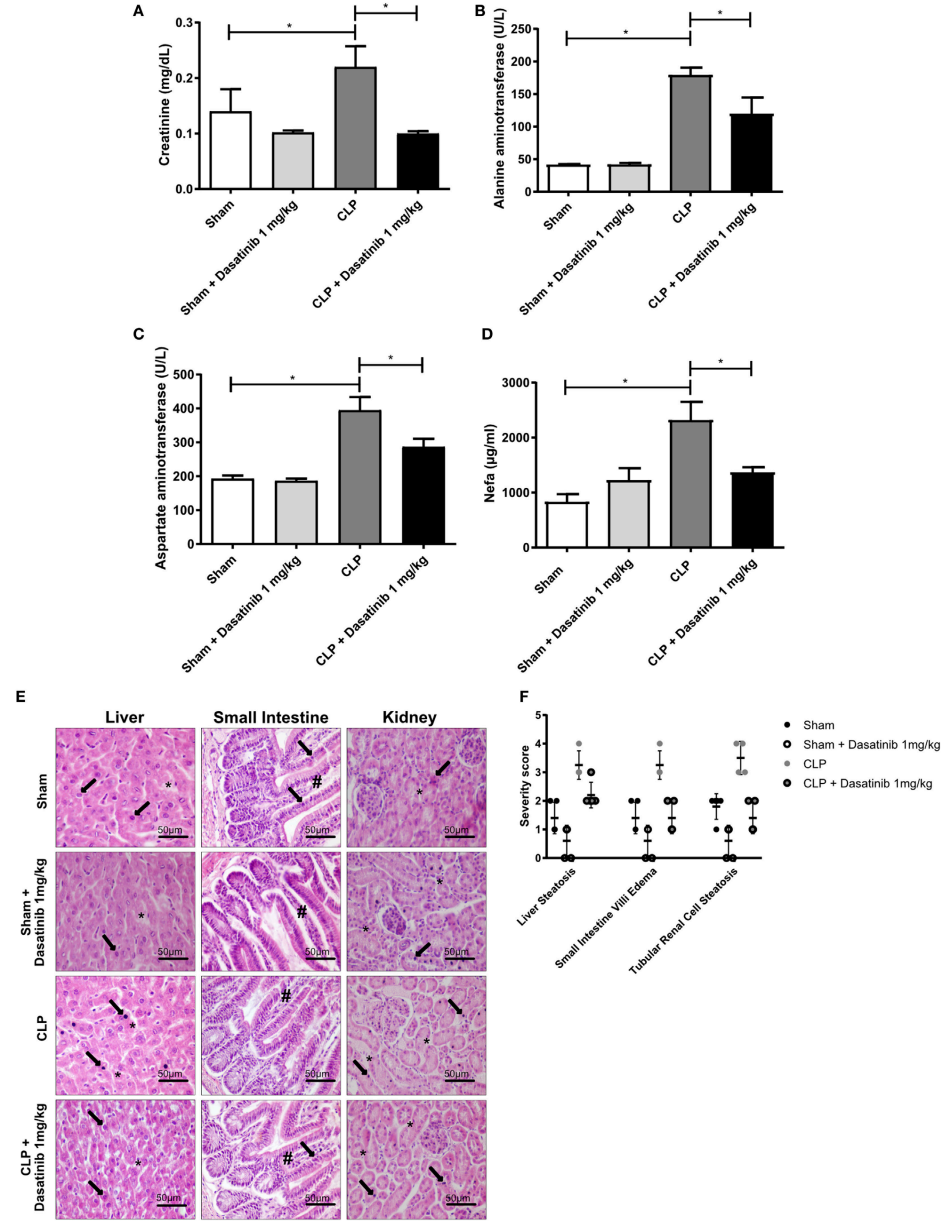
Low dosage of dasatinib decreases organ dysfunction in septic animals. Swiss mice were submitted cecal ligation and puncture (CLP). Sham-treated animals were used as control. The animals were treated with dasatinib at 1 mg/kg 30 min before and 6 h after CLP. Blood was collected 24 h after CLP procedure, and organs were harvested for HE staining. **(A)** Creatinine, **(B)** alanine, **(C)** aspartate aminotransferase, and **(D)** non-esterified fatty acid (NEFA) were analyzed. Data are presented as mean ± SEM. **(E)** Optical microscopy of liver, small intestine, and kidney. In the CLP group, liver hepatocytes around of centrilobular vein and tubular renal cells exhibit apoptosis (arrows) and diffuse vacuolization in the cytoplasm by accumulation of fat (asterisks), thus characterizing liver and kidney steatosis; the small intestine show apoptosis of enterocytes (arrows) and prominent edema of the villi (#). After dasatinib treatment, the integrity of liver hepatocytes, small intestine villi, and tubular renal cells are restored with reduction in apoptosis and steatosis score similar to Ssam and Sham animals treated with dasatinib. Scale bar is 50 µm. **(F)** Injury score with severity analyses of microscopically visible organ damage. The score ranges from 0 to 4 where 0 means no injury and 4 maximum injuries. Statistical analysis: one-way ANOVA followed by Tukey *P < 0.05. The number of animals per group range from 4 to 10.

To evaluate the protective effect of dasatinib treatment in septic animals in more detail, we analyzed the impact of dasatinib treatment in sepsis-induced organ dysfunction. We measured plasma biological markers for kidney (creatinine) and liver (aspartate and alanine aminotransferase), and lipotoxicity (NEFA). All CLP animals displayed significantly increased levels of creatinine, aspartate aminotransferase, NEFA, indicating severe organ damage caused by CLP induced sepsis (Figures 5A–D). Dasatinib treatment at 1 mg/kg lowered these levels in CLP mice indicating that dasatinib can partly rescue sepsis-induced organ damage. Reduced plasma levels of albumin and glucose after CLP could not be rescued by dasatinib (Figures S2A,B in Supplementary Material).

In agreement with systemic biochemical markers of organ dysfunction, histological alterations were detected in the liver (first column), the small intestine (second column), and the kidney (third column) of septic mice, compared to control group (Figure 5E). CLP induced liver and kidney steatosis and edema in small intestine villi. However, treatment with 1 mg/kg dasatinib prevented these alterations. Histological changes were scored from 0 (without alterations) to 4 (more extensive lesions) and we could detect an overall decrease in the severity of the organ damage in CLP mice treated with dasatinib to the score levels of CLP mice (Figure 5F). In the omentum, we found some neutrophil infiltration due to the inflammatory reaction induced by surgery in sham animals. Dasatinib treatment did not affect omentum morphology or leukocyte infiltration in sham animals (Figure S2C in Supplementary Material). In septic mice, the omentum seems to be liquefied due to the intensity of the acute inflammatory response taking place in the peritoneal cavity. In dasatinib-treated animals, the omentum histology was similar to the sham conditions, reinforcing the protective role of dasatinib in CLP induced sepsis.

### Dasatinib Treatment Impaired the Number of Leukocytes in the Peritoneal Cavity and Decreased Concentration of Inflammatory Mediators

**Figure 6:**
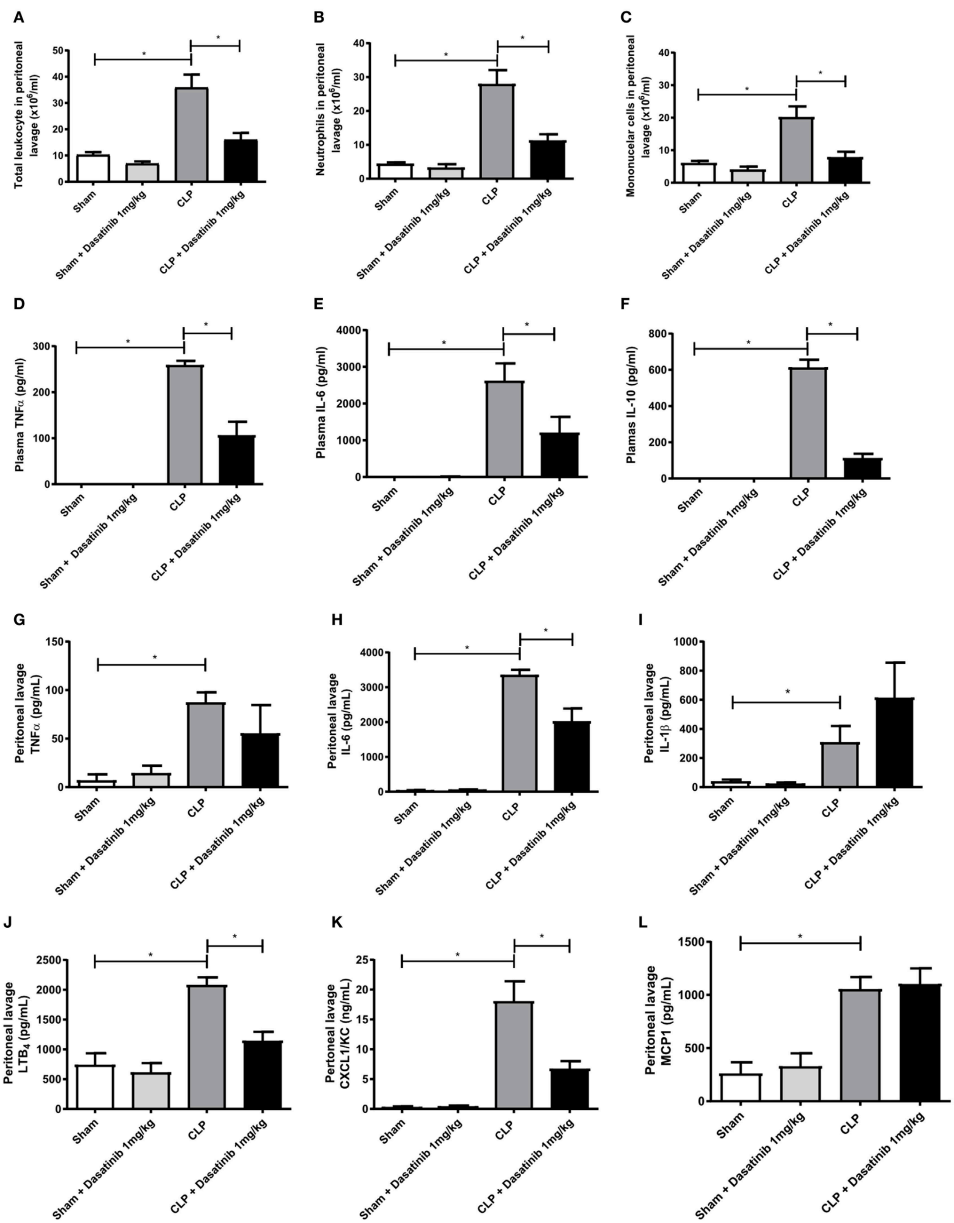
Dasatinib treatment results in fewer peritoneal leukocytes along with decreased amounts of inflammatory markers. Swiss mice were submitted cecal ligation and puncture (CLP). Sham-treated animals were used as control. The animals received dasatinib at 1 mg/kg 30 min before and 6 h after CLP. Cells were collected 24 h after CLP procedure by peritoneal lavage to assay total and differential counts. Data are presented as mean ± SEM. **(A)** Total leukocyte count, **(B)** mononuclear cell count, and **(C)** neutrophil cell count are shown. Cytokines were measured by ELISA and LTB4 by EIA. **(D–F)** Display plasma levels of tumor necrosis factor (TNF)-α **(D)**, interleukin (IL)-6 **(E)**, and IL-10 **(F)**, **(G–L)** display peritoneal lavage values of TNF-α **(G)**, IL-6 **(H)**, IL-1β **(I)**, LTB4 **(J)**, CXCL1 **(K)**, and MCP1 **(L)** Statistical analysis: one-way ANOVA followed by Tukey *P < 0.05, **P < 0.01, and ***P < 0.001. The number of animals per group range from 6 to 18.

We next investigated the effect of dasatinib treatment in leukocyte accumulation and inflammatory mediators in more detail. For this purpose, we analyzed cell accumulation in the peritoneal cavity 24 h after CLP (Figures 6A–C). As expected, septic animals had higher numbers of mononuclear cells and neutrophils in the inflamed peritoneal cavity as compared to sham-treated animals (Figures 6A–C). Treatment with dasatinib (1 mg/kg) significantly lowered both mononuclear cell and neutrophil accumulation in the peritoneal cavity (Figures 6B,C). This finding is in accordance to our previous data of reduced leukocyte extravasation in inflamed cremaster muscle tissue after dasatinib treatment.

Additionally, we analyzed the effect of dasatinib (1 mg/kg) on cytokine, and chemokine production. We measured plasma levels of TNF-α, IL-6, and IL-10 in dasatinib-treated animals 24 h after CLP and compared them to sham-treated mice. Septic animals presented elevated levels of all measured cytokines (Figures 6D–F). Animals treated with dasatinib at 1 mg/kg dose presented significantly lower levels of all cytokines indicating a reduced extend of inflammation. We also measured the levels of cytokines, chemokines, and lipid mediators in the peritoneal lavage of septic mice. The levels of TNF-α and IL-6 were increased in septic mice and treatment with dasatinib decreased their levels (Figures 6G,H). Dasatinib administration did not affect IL-1β levels (Figure 6I). Septic mice also displayed increased peritoneal levels of LTB_4_ and CXCL1/KC. Likewise 1 mg/kg dasatinib decreased LTB_4_ and CXCL1/KC levels (Figures 6J,K), while MCP1 levels remained at the same levels as in the CLP group (Figure 6L).

### Dasatinib Inhibited Bacterial Growth and Bacterial Spreading in Septic Mice

**Figure 7:**
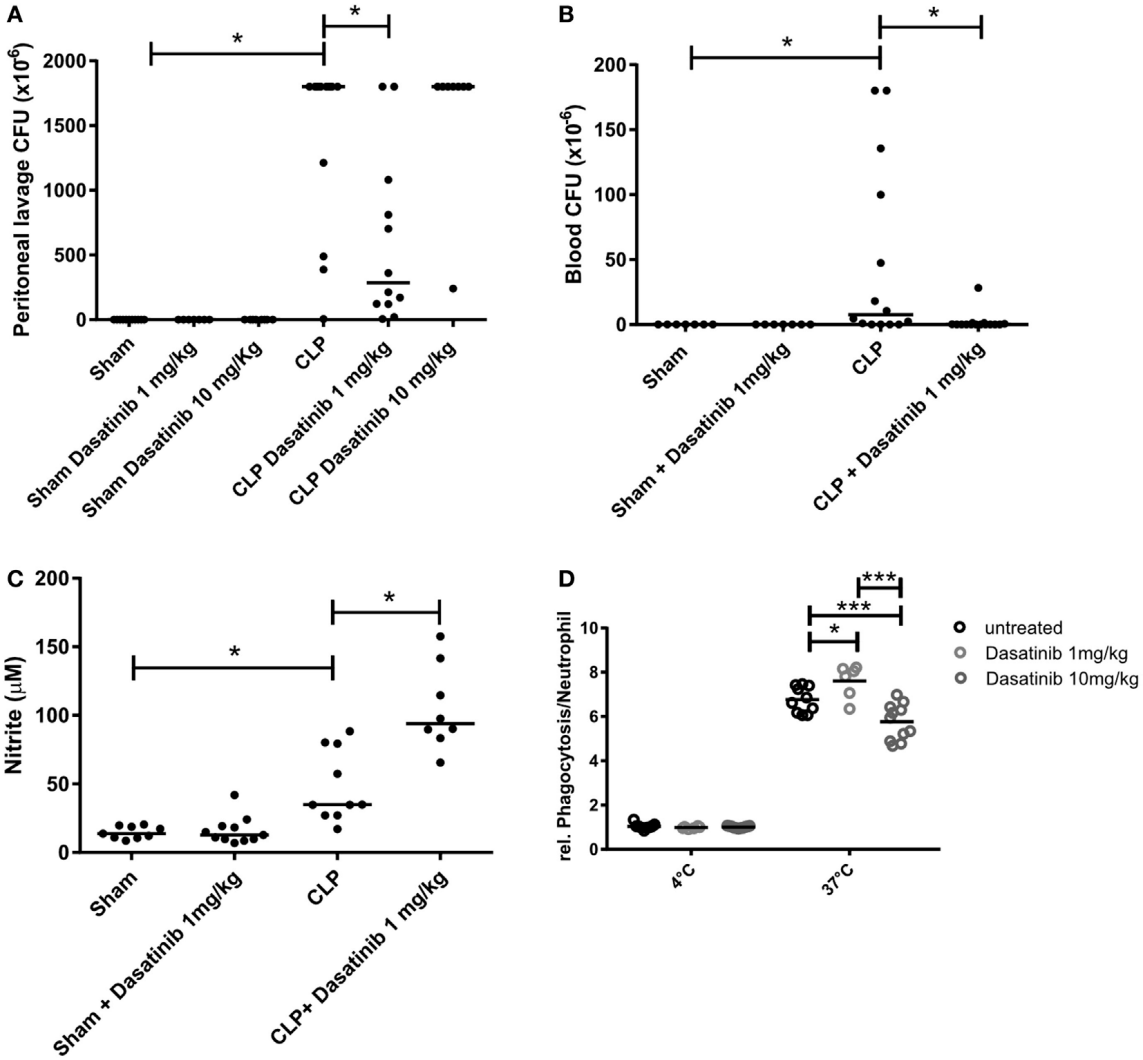
Dasatinib treatment decreased colony-forming unit (CFU) counts and simultaneously enhances neutrophil function. *Swiss* mice were submitted cecal ligation and puncture (CLP). Sham-treated animals were used as control. The animals received dasatinib at 1 mg/kg 30 min before and 6 h after CLP. Data are presented as mean ± SEM. The CFU was determined 24 h after CLP in **(A)** the peritoneal lavage fluid and **(B)** blood. **(C)** Cell-free supernatant from peritoneal lavage of animals was collected 24 h after CLP, and nitrite levels was measured by Griess reaction. **(D)** Relative neutrophil phagocytosis was tested in blood neutrophils from dasatinib or vehicle treated cells using pHrodo *E coli* bioparticles. Statistical analysis: one-way ANOVA followed by Tukey *P < 0.05, **P < 0.01, and ***P < 0.001. The number of animals per group range from 6 to 15.

About 60–70% of patients with sepsis have positive blood cultures, most of them are Gram-negative bacteria (5, 44). In our model, we have a mixed infection with both Gram-negative and Gram-positive bacteria, detected in the peritoneal fluid from septic animals. Interestingly, 1 mg/kg dasatinib significantly reduced CFU counts in the peritoneal fluid (Figure 7A). At 10 mg/kg, however, dasatinib-treated CLP animals showed higher CFU numbers compared to CLP (Figure S3A in Supplementary Material). We also evaluated CFU formation in distal organs to assess bacterial translocation and the ability of the organism to fight infection. We could detect high numbers of CFUs in all analyzed organs of CLP animals. Again 1 mg/kg dasatinib successfully prevented bacterial translocation to the blood (Figure 7B), lung, spleen, kidney, and liver (Figures S3B–E in Supplementary Material).

### Dasatinib Treatment Enhanced Neutrophil Functionality

Neutrophils phagocytose microbes, produce ROS, release antimicrobial factors and form NET as part of their arsenal to fight invading organisms (45). In order to explore why treatment with dasatinib was able to decrease CFU numbers despite reducing the numbers of neutrophils at the site of infection, we investigated the effect of dasatinib on the ability of neutrophils to kill bacteria. To evaluate the effect of dasatinib on NET formation, we measured extracellular double-strand DNA *via* fluorimetry in septic animals with and without dasatinib. As shown in Figure S3F in Supplementary Material, the CLP group showed an increase in extracellular DNA content compared to sham animals. Interestingly, treatment with dasatinib did neither alter extracellular DNA levels nor did the disassembling of NETs by DNAse interfere with the ability of dasatinib to decrease CFU numbers (Figure S3G in Supplementary Material).

Next, we checked the production of nitrite as readout for NO production in the peritoneal cavity after treatment with 1 mg/kg dasatinib. NO and superoxide generate antimicrobial molecules called reactive nitrogen species that act together with ROS in damaging cells and microbes (46, 47). Our results show that dasatinib increased local nitrite production during CLP, which contributes to bacterial clearance (Figure 7C). Next we examined the effect of dasatinib treatment on neutrophils phagocytosis. Mice were treated with 1 mg/kg dasatinib and neutrophils phagocytosis was determined in whole blood by flow cytometry. Interestingly, compared to untreated animals, animals treated with dasatinib (1 mg/kg) showed increased neutrophil phagocytosis (Figure 7D), suggesting a potential enhancing effect on the ability of neutrophil to clear bacteria. Also here we encountered a dose-dependent effect, because treatment with 10 mg/kg dasatinib decreased the ability of neutrophils to phagocytose.

## Discussion

Sepsis is one of the leading causes of morbidity and mortality in Intensive Care Units, and is associated with increased health-care costs (48, 49). This is complicated by the rise of drug-resistant microorganisms, a growing elderly population, and an increased incidence of immunosuppression (50–54). The failures of anti-toll-like receptor 4 antibody, recombinant activated protein C, and anti-TNF-α therapies in clinical trials require a rethinking of sepsis’ pathophysiology and therapeutic strategies (8, 55–60). Systematic approaches, such as presented here, could fuel the discovery of promising immunosuppressive or anti-inflammatory drugs that aim at multifunctional targets such as Src family kinases.

Src family kinases play critical roles in a whole variety of pathologies including cancer; in addition, it was shown that Src is involved in inflammation-related signaling pathways (61). Dasatinib is a type I ATP-competitive protein kinase inhibitor (62). SFK-inhibitors affect signaling pathways and downmodulate the inflammatory response. Nevertheless, the current dose of dasatinib (100 mg daily in human) used to treat some leukemia does not induce severe immunosuppression (63). In the present work, we used lower doses of dasatinib (1 and 10 mg per kg). We chose these doses because initial pharmacokinetic experiments showed that dasatinib at 1 mg per kg yields plasma concentrations that remained above the critical concentration to inhibit SFKs.

It is currently accepted that it is not the insult *per se*, but the host’s response, that determines severity and outcome in sepsis (4). Therefore, the immunosuppressive action of dasatinib may affect the response against infectious agents and it is expected that high-dose favors the progression of infection with deleterious effects to the host as shown for *Pneumocystis jiroveci* pneumonia (63). On the contrary, lower doses may modulate the immune response affecting and/or preventing tissue damage resulting from host immune response or cellular hyperactivation. In fact, we show here that dasatinib at 10 mg/kg is deleterious to the host fueling infection progression. In contrast, the lower dose of dasatinib (1 mg/kg) showed promising results improving the animal clinical condition and increasing survival. The higher dasatinib dose may inhibit other kinases and proteins impacting on ability of the host to fight the infection effectively because of its potent anti-inflammatory effect. Lower dose of dasatinib decreases the neutrophil migration but does not abrogate it. So the fewer neutrophils that reach the peritoneum remain effective on killing the bacteria and restrain the infection.

Septic patients present systemic inflammation with exacerbated cytokine production, and increased cell migration to the site of infection or sterile inflammation, as shown with CLP mice in this publication. Trafficking of myeloid leukocytes to the site of inflammation is linked to the generation of an appropriate inflammatory environment (20). In this regard, LTB_4_ is a potent chemotactic agent for neutrophils (64). Its levels increase 24 h after CLP and treatment with dasatinib (1 mg/kg) reduced the levels of LTB4. Dasatinib also decreased the levels of CXCL1/KC, another potent chemo attractant to neutrophils. CXCL1/KC is released by resident macrophages and mediates neutrophil accumulation induced by LPS (65).

Src family tyrosine kinases are important components of the signaling pathways initiated by the TLRs (critical for cytokine production) and many cytokines, such as TNF, use Src family kinases in their own signaling pathways (17, 66). In our model, dasatinib reduced the levels of all measured cytokines confirming data from the literature (25, 67), except IL-1β. Impairing TLR4-related signaling pathway inhibits cytokine production including both TNF and IL-10 (68). IL-1β requires the cleavage of the pro-form (pro-IL-1β) by caspase-1 into its biologically active form (69). However, we cannot exclude that IL-1β detected here may reflect the release of already present IL-1β in the cell which would suggest that the release of IL-1β is independent of Src kinases (70) while the other measured cytokines are upregulated by SFK-dependent transcriptional activity, reinforcing the key role of SFK in cytokine production in infectious disease.

Activated neutrophils are able to generate reactive oxygen species, release NETs, increase phagocytosis, and produce nitric oxide (45). We further elucidated the role of tyrosine kinases in these processes and found that dasatinib did not affect NET formation. In contrast, nitric oxide generation was increased in septic mice treated with dasatinib strengthening its potent bactericidal activity at the local level (71, 72).

Phagocytosis is a prime mechanism of bacterial killing. Src-family kinase-deficient leukocytes are less effective than wild-type cells at mediating phagocytosis (73, 74). On the other hand macrophages lacking Src family members Hck, Fgr, and Lyn showed phagocytosis mediated by Fcγ Receptor (75), so phagocytosis obviously can happen independently of neutrophil-expressed SFKs. Interestingly, our experiments revealed that 1 mg/kg of dasatinib increased phagocytotic activity of blood neutrophils compared to control neutrophils, while higher doses (10 mg/kg dasatinib) decreased phagocytosis. The mechanism for this dose-dependent effect of dasatinib on phagocytotic activity in neutrophils is currently unclear and needs further investigations.

Patients with severe sepsis symptoms display metabolic dysfunction with elevated levels of plasma NEFA and lower levels of albumin (76, 77). NEFA activates TLRs boosting cytokine production (78), induce cell death (79), and inhibit sodium potassium ATPase in several organs, including the lungs (80). In addition, the inhibition of lipogenesis or the increase in lipid oxidation reduces levels of free fatty acids, TNF-α, and IL-6, and reduces liver injury improving survival in sepsis (81, 82). Accordingly, the observed decrease in NEFA levels in the plasma of dasatinib-treated mice might at least in part be responsible for their improved organ function.

Regardless of their clinical potential in septic patients, our study demonstrates that the use of SFK inhibitors needs to be tightly controlled to keep the fine balance between overtreatment with uncontrolled bacterial growth in an immuno-compromised organism and ineffective treatment leading to a hyper-inflammatory response of the host immune system. Keeping this balance at an optimal level will certainly be a challenge and require intensive monitoring. In view of the fact that great efforts have been made to develop tyrosine kinases inhibitors for the therapy of inflammatory diseases (83), their use might open new doors in modulating the inflammatory response during sepsis and, therefore, improve the outcome of patients suffering from this life-threatening syndrome.

## Ethics Statement

Animal housing conditions and experimental procedures con-formed to institutional regulations and were in accordance with the National Institute of Health guidelines on animal care. The Institutional Animal Welfare Committee approved all procedures described here under license number 002-08, LW36/10, and L15/2015. The animal experiments were approved by the Regierung von Oberbayern, Germany (AZ 55.2-1-54-2531-80-76/12). Both Institutions follow the ARRIVE guidelines (Animal Research: Reporting of In Vivo Experiments) originally published in 2010 (84).

## Author Contributions

Conceptualization: CG-d-A, IR, AS, PR, MS, and HC-F-N. Data curation: CG-d-A, IR, AS, AF, AK, CC, SK, TE, JS, GO, VC, GS, EC, RM, BW, MS, PR, and HC-F-N. Formal analysis: C-G-d-A, IR, AS, AF, AK, and CC. Funding acquisition: HC-F-N, PR, AS, CG-d-A, MS, BW, AM, and IM-P. Investigation: CG-d-A, IR, AS, AF, AK, CC, SK, TE, JS, GS, EC, and RM. Project administration: CG-d-A, IR, AS, CC, PR, BW, PR, MS, and HC-F-N. Supervision: AS, AM, IM-P, PR, MS, and HC-F-N. Validation: CG-d-A, IR, AS, CC, BW, PR, MS, and HC-F-N. Visualization: CA, IR, AS, CC, MS, and HN. Writing—original draft preparation: CG-d-A, IR, AS, MS, and HN. Writing—review and editing: CA, IR, AS, AF, AK, CC, SK, TE, JS, GO, VC, GS, EC, RM, AM, IM-P, BW, PR, MS, PR, and HC-F-N.

## Conflict of Interest Statement

The authors declare that the research was conducted in the absence of any commercial or financial relationships that could be construed as a potential conflict of interest.
