# Supplementary material for: The Yin and Yang of Tyrosine Kinase Inhibition During Experimental Polymicrobial Sepsis
Source: Front Immunol. 2018 Apr 30;9:901. doi: 10.3389/fimmu.2018.00901 (PMC5936983; doi:10.3389/fimmu.2018.00901)
Supplement: Supplementary file 1 [file data_sheet_1.DOCX]

Supplementary Material

The yin and yang of Src family tyrosine kinase inhibition during experimental polymicrobial sepsis

**Cassiano Felippe Gonçalves-de-Albuquerque1,2,7,&, Ina Rohwedder2&, Adriana Ribeiro Silva1, Alessandra Silveira Ferreira1, Angela R.M. Kurz1,2,3, Céline Cougoule5, Sarah Klapproth2, Tanja Eggersmann2, Johnatas D Silva4, Gisele Pena de Oliveira4, Vera Luiza Capelozzi7, Gabriel Gutfilen Schlesinger1, Edlaine Rijo Costa6, Rita de Cassia Elias Estrela Marins5,10, Attila Mócsai9, Isabelle Maridonneau-Parini5, Barbara Walzog2, Patricia Rieken Macedo Rocco4, Markus Sperandio2+, Hugo Caire de Castro-Faria-Neto1#*+* Correspondence:** Corresponding authors: Hugo Caire de Castro-Faria-Neto, hugocfneto@gmail.com, Laboratório de Imunofarmacologia, Instituto Oswaldo Cruz, FIOCRUZ, 21040-900 Rio de Janeiro, RJ, Brazil and Markus Sperandio, Markus.Sperandio@lmu.de, Walter Brendel Centre, Ludwig Maximilians University München, Germany.

## Supplementary Figures


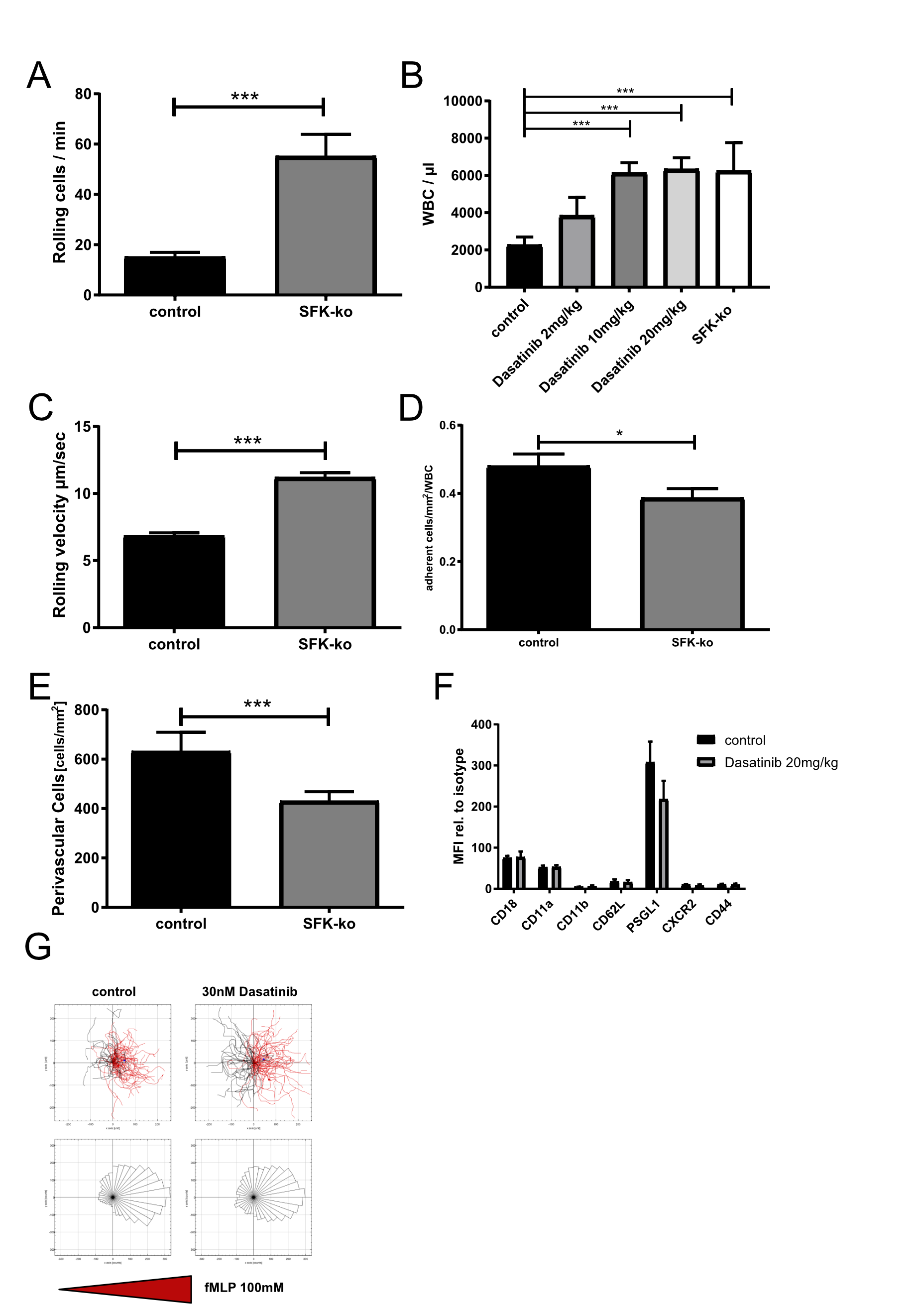


**Supplementary Figure 1:** Leukocyte rolling, adhesion and transmigration in SFK-ko animals. Data are presented as mean ± SEM. *P < 0.05 and ***P < 0.001. (A) Number of rolling cells / min in SFK-ko animals compared to control animals are shown. (B) Average WBC count in control, or 2 mg/kg, 10 mg/kg or 20 mg/kg Dasatinib treated Lyz2GFP mice and SFK-ko mice 2h after rmTNF-α application. (C) Rolling velocity of SFK-ko leukocytes compared to control is displayed (D) Adhesion efficiency of SFK-ko and control leukocytes was calculated by the number of adherent cells/ mm^2^ normalized to the total WBC count. (E) Total number of extravasated cells/mm^2^ in muscle tissue in close proximity to a vessel in SFK-ko and control animals. (F) FACS analysis of leukocyte surface markers. Mean fluorescence intensities relative to isotype control are shown. Data are presented as mean ± SEM. (G) Three-dimensional migration PMNs with or without Dasatinib towards a fMLP gradient in a collagen gel matrix. Representative single cell migration tracks and rose plots are displayed. Arrow indicates orientation of gradient. At least 80 cells were analyzed for each strain. ***P < 0.0001


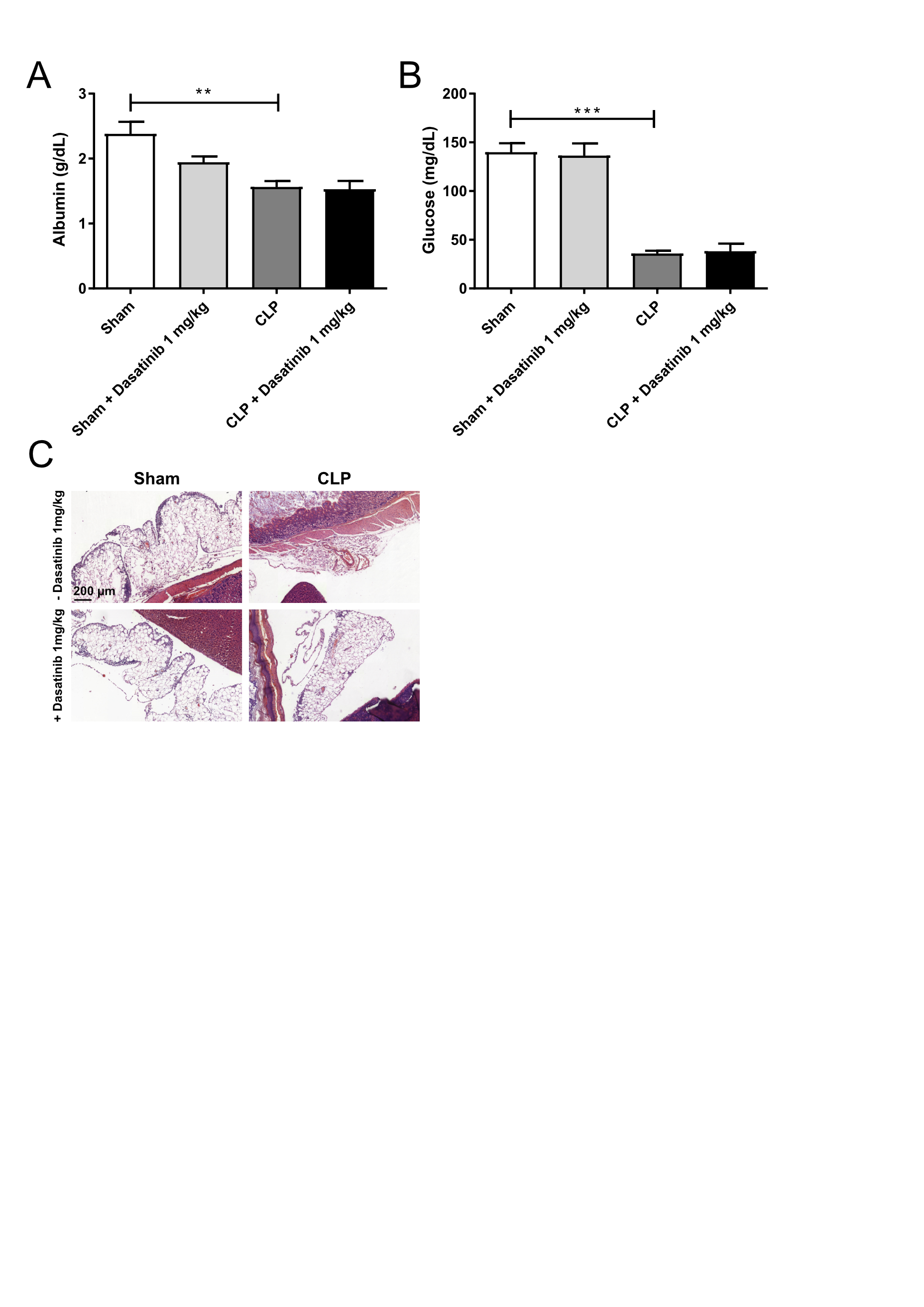
**Supplementary Figure 2:** No alteration in albumin and glucose levels in septic animals after treatment with 1mg/kg Dasatinib. Swiss mice were submitted to CLP and sham treated animals were used as control. The blood was collected 24 h after CLP procedure. The animals were treated with Dasatinib at 1 mg/kg 30’ before and 6 h after CLP. (A) Albumin and (B) glucose levels were analyzed. Data are presented as mean ± SEM. (C) HE staining of paraffin sections of the peritoneal region. In addition to the omentum, the stomach, pancreas and spleen are displayed in higher magnification. Statistical analysis: one-way ANOVA followed by Tukey **P < 0.01 and ***P < 0.001. The number of animals per group range from 5 to 10.

**
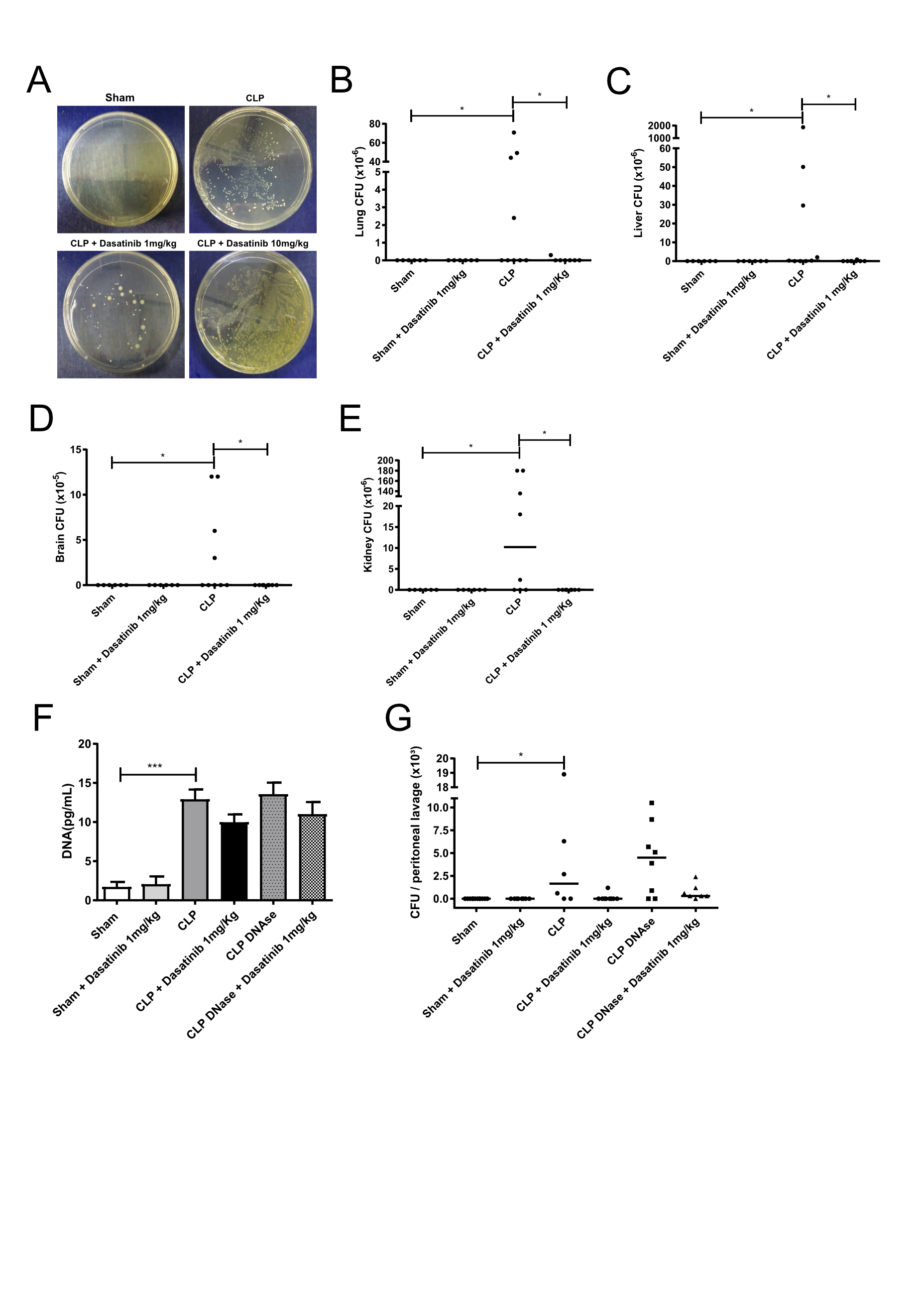
**

**Supplementary Figure 3:** Effect of Dasatinib on CFU counts in various organs and NET formation. Swiss mice were submitted CLP. Sham treated animals were used as control. The animals received Dasatinib at 1 mg/kg 30’ before and 6 h after CLP. (A) Representative picture of bacterial colonies on agar plate grown for 18 h. The CFU was determined 24 h after CLP in (B) lung, (C) spleen, (D) kidney and (E) Liver. Data are presented as mean ± SEM. For analysis of NET formation, mice were treated with Dasatinib at 1 mg/kg 30 min before CLP. Some animals additional received DNase treatment 1 h after CLP (0.5 mg/kg, i.v.). DNA quantification and CFU numbers were performed in the peritoneal lavage obtained 3 hours after surgery (F and G). Data are presented as mean ± SEM. *P < 0.05. Statistical analysis: Student t Test for B, C, D and E and one-way ANOVA followed by Tukey *P < 0.05 and ***P < 0.001. The number of animals per group range from 6 to 12.
